# A Rare Case of Anti‐Yo Antibody Positive Paraneoplastic Neurologic Syndromes With EGFR Mutation Positive Non‐Small Cell Lung Cancer

**DOI:** 10.1002/rcr2.70560

**Published:** 2026-03-16

**Authors:** Nobuhiro Okagaki, Masakuni Ueyama, Shoko Fujimoto, Shigenari Iwagaki, Hiroto Sakamoto, Yuma Tanaka, Tsukasa Nakanishi, Satoshi Nakamura, Kazuki Matsumura, Naoya Ikegami, Yusuke Kaji, Seishu Hashimoto, Eisaku Tanaka, Yoshio Taguchi, Akihisa Fukunaga, Toshihiko Suenaga, Naomi Kanamori, Takeshi Kubo, Takashi Hajiro

**Affiliations:** ^1^ Department of Respiratory Medicine Tenri Hospital Tenri City Japan; ^2^ Department of Neurology Tenri Hospital Tenri City Japan; ^3^ Department of Pathology Tenri Hospital Tenri City Japan; ^4^ Department of Radiology Tenri Hospital Tenri City Japan

**Keywords:** anti‐Yo antibody, EGFR mutation, non small cell lung cancer, paraneoplastic neurologic syndromes, rapidly progressive cerebellar syndrome

## Abstract

Paraneoplastic neurologic syndromes (PNS) associated with anti‐Yo antibody typically occur in females with ovarian or breast cancer, and their occurrence in lung cancer is rare. We report the first case of anti‐Yo antibody positive PNS in a patient with EGFR mutation‐positive non‐small cell lung cancer (NSCLC). A 74‐year‐old male presented with acute vertigo and progressive cerebellar ataxia. Anti‐Yo antibody was positive, and transbronchial biopsy confirmed papillary adenocarcinoma with EGFR exon 21 L858R mutation. Brain MRI also revealed multiple metastases, and cerebrospinal fluid cytology was positive for malignant cells. Despite the presence of brain metastases and leptomeningeal metastasis, neurological symptoms were attributed primarily to anti‐Yo PNS based on clinical presentation. Chemotherapy was discontinued due to interstitial lung disease, but subsequent steroid therapy resulted in modest improvement of ataxic symptoms. This case suggests that NSCLC presenting with anti‐Yo PNS may harbour EGFR mutations.

## Graphical abstract

We report the case of Anti‐Yo antibody positive paraneoplastic neurologic syndromes (PNS) with EGFR mutation positive non‐small cell lung cancer (NSCLC). Although anti‐Yo PNS is rare in NSCLC, there is a possibility of EGFR gene mutation positivity.

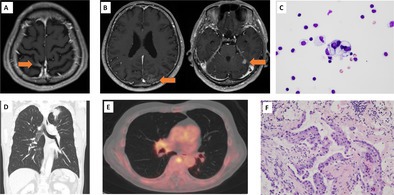

## 1 Introduction

Anti‐Yo (PCA‐1) antibody positive paraneoplastic neurologic syndromes (PNS) predominantly affect females with pelvic or breast cancer [1]. It rarely occurs in males, mostly associated with gastrointestinal cancer [2]. We present a rare case of anti‐Yo PNS with EGFR‐mutated non‐small cell lung cancer (NSCLC).

## 2 Case Report

**FIGURE 1:**
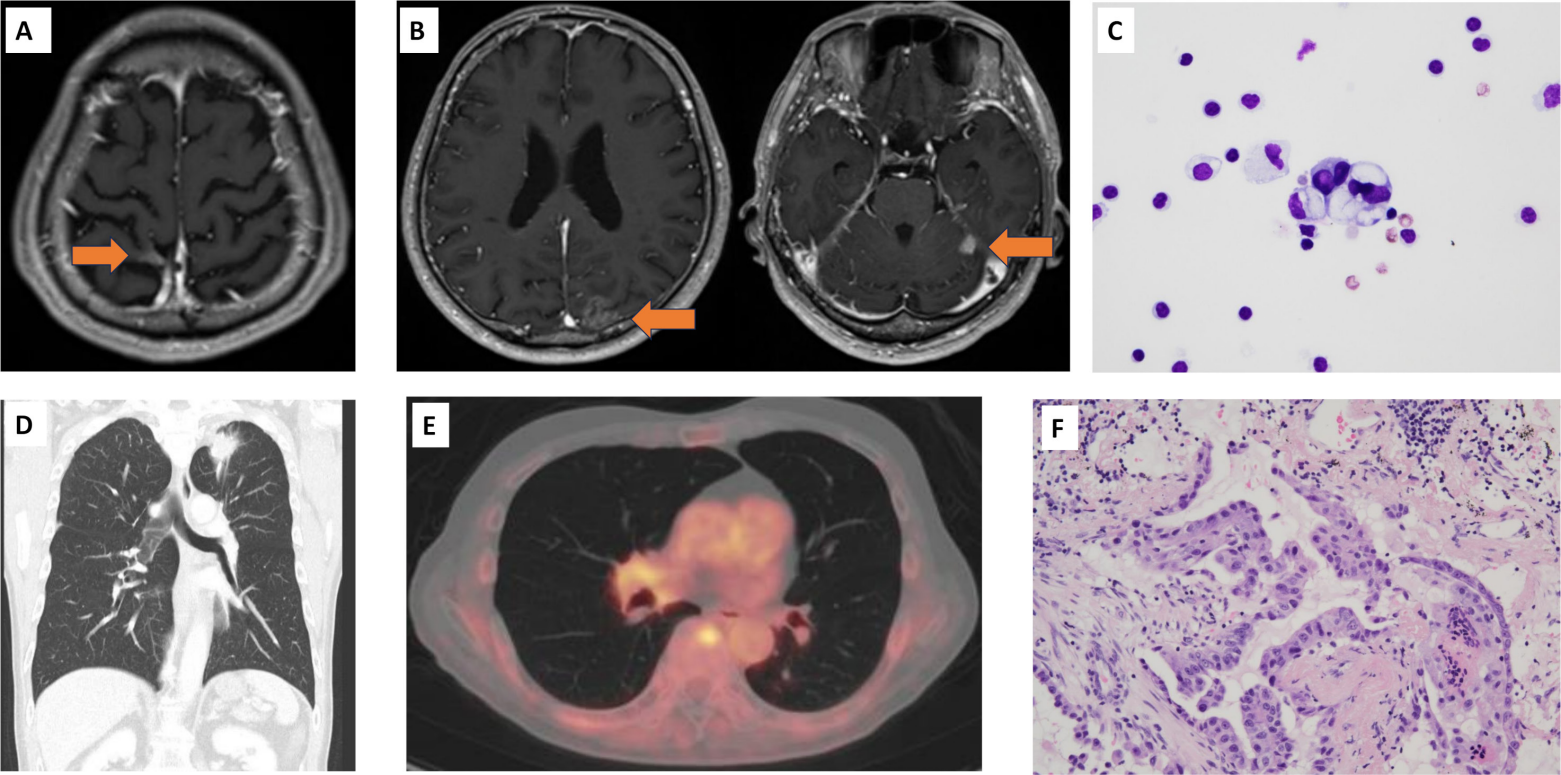
Radiological and pathological images. An enhanced head magnetic resonance imaging demonstrated leptomeningeal enhancement suggestive of leptomeningeal dissemination (A), as well as multiple brain metastases (B). The metastatic lesions were observed in the left frontal lobe, occipito‐parietal lobe, and left cerebellar hemisphere. There were no lesions in the vermis, which is atypical as a cause of ataxia; Cerebrospinal fluid cytology was positive for malignant cells. Haematoxylin and eosin stain, 40× (C); Chest computed tomography revealed a left upper lobe nodule 3.2 cm in size (D); FDG‐PET shows elevated FDG uptakes in the left upper lobe nodule and the eighth thoracic vertebral body (E); The biopsy specimen of the left upper lobe lesion revealed papillary adenocarcinoma, Haematoxylin and eosin stain, 100× (F).

**FIGURE 2:**
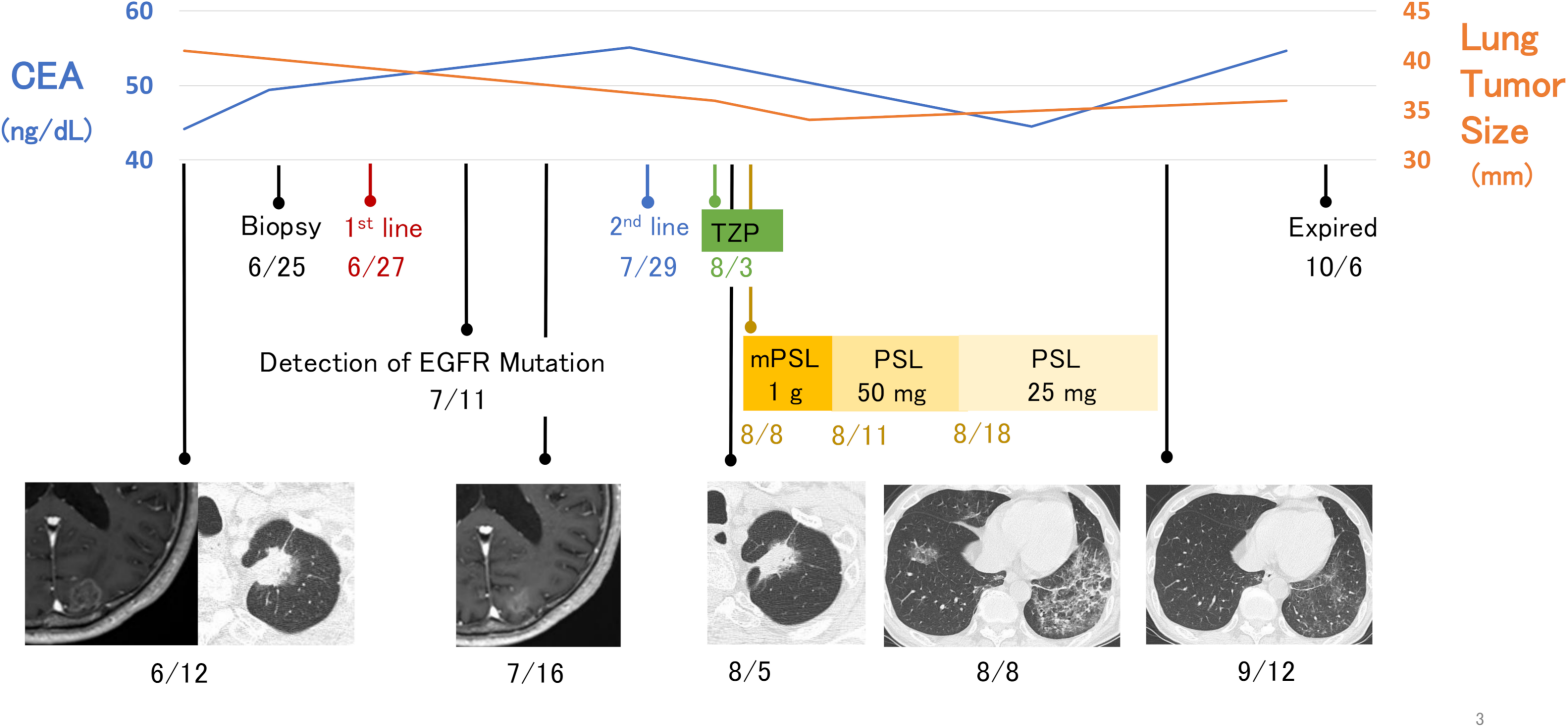
Clinical course and management. Abbreviation: ABCP, Atezolizumab‐bevacizumab‐carboplatin‐paclitaxel; CBDCA, carboplatin; CEA, carcinoembryonic antigen; CRP, C‐reactive protein; mPSL, methylprednisolone; PEM, pemetrexed; PSL, prednisolone; TZP, piperacillin/tazobactam.

A 74‐year‐old man with treated pulmonary tuberculosis and a previous 12‐pack‐year smoking history experienced acute vertigo and presented to our hospital 1 month later. He had marked cerebellar signs with a tendency to lean leftwards when walking, instability during step‐over gait and left‐leg standing. Reflexes, sensation and coordination were intact. His power was Medical Research Council grade 5 in all major muscle groups. Enhanced head magnetic resonance imaging (MRI) demonstrated multiple brain lesions (Figure 1A,B). Cerebrospinal fluid (CSF) analysis revealed an opening pressure of 15.5 cmH₂O, a nonerythroid cell count of 10 cells/μL, and a protein level of 56 mg/dL. Cytological examination of the CSF revealed malignant cells (Table 1, Figure 1C). The serum anti‐Yo antibody was positive [2+] on immunoblot/line assay (Table 1). Chest computed tomography revealed a 3.2‐cm left upper lobe nodule (Figure 1D). FDG‐PET showed increased uptake in the lung lesion and the eighth thoracic vertebral body (Figure 1E). Bronchoscopic biopsy revealed papillary adenocarcinoma, staged as clinical T3N0M1c (stage IVb) (Figure 1F), meeting the diagnostic criteria for probable PNS [3]. Despite the presence of leptomeningeal metastasis and multiple brain metastases, neurologists concluded that the neurological symptoms, consisting mainly of rapidly progressive ataxia, were derived from Anti‐Yo PNS. Immunosuppressive therapy for PNS was not recommended due to prior tuberculosis and previous HBV infection pattern. As neurological symptoms progressed rapidly, chemotherapy was initiated immediately after diagnosis. Atezolizumab‐bevacizumab‐carboplatin‐paclitaxel (ABCP) therapy was initiated, and subsequently an EGFR Exon 21 L858R mutation was detected by Oncomine DxTT. During the first course of chemotherapy, cerebellar symptoms progressed, causing difficulty walking. Chest CT showed a reduction in pulmonary nodules, and MRI indicated a partial reduction in some brain metastases. Despite the efficacy against brain metastasis, neurological symptoms worsened, suggesting the influence of PNS. Furthermore, oedema developed in the left lower leg. Venous ultrasonography revealed deep venous thrombosis, and edoxaban was initiated. ABCP therapy was deemed inadequate for disease control, leading to initiation of osimertinib in combination with pemetrexed and carboplatin (Figure 2). On Day 7 of second‐line treatment, the patient developed fever and dyspnea, and chest CT showed bilateral infiltrates. Antibiotics proved ineffective, and glucocorticoid therapy was initiated following the diagnosis of grade two interstitial lung disease (ILD), necessitating discontinuation of chemotherapy. The performance status (PS) also worsened to 3 because of progressive ataxia and dyspnea. He received methylprednisolone (1000 mg) for 3 days, followed by oral prednisolone at 1 mg/kg/day. Subsequently, the dyspnea and infiltrative shadows improved and ataxic symptoms also showed modest improvement, suggesting a possible therapeutic effect on the PNS. Although the PS temporarily improved from 3 to 1, it eventually deteriorated due to the development of ataxia, dysphagia and disorientation during the treatment interruption. The patient transitioned to best supportive care and died of aspiration pneumonia 2 months after initiation of second‐line therapy.

**TABLE 1:** Initial haematological and CSF data.

Blood analysis						Serum paraneoplastic antibody panel		CSF analysis		
Red blood cells	4.16	×10^6^/μL	C‐reactive protein	0.05	mg/dL	Anti‐AMPH Antibody	(−)	Intracranial pressure	15.5	cmH_2_O
Haemoglobin	14.1	g/dL	KL‐6	635	U/ml	Anti‐CV Antibody	(−)	Protein	56	mg/dL
Platelets	18.9	×10^4^/μL	Folic Acid	13	ng/mL	Anti‐ PNMA2 Antibody	(−)	Glucose	42	mg/dL
White blood cells	6.33	×10^3^/μL	Vitamin B12	1.3	ng/mL	Anti‐Ri Antibody	(−)	Erythroid	< 200	cells/μL
D‐dimer	2.3	μg/dL	Vitamin B1	33	ng/mL	Anti‐Yo Antibody	(2+)	Nonerythroid	10	cells/μL
BUN	15.0	mg/dL	CEA	44.1	ng/mL	Anti‐Hu Antibody	(−)	Neutrophils	0.0	%
Creatinine	0.77	mg/dL	CYFRA	3.5	ng/mL	Anti‐recoverin Antibody	(−)	Lymphocytes	82.5	%
Glucose	99	mg/dL	proGRP	49.4	pg/mL	Anti‐SOX1 Antibody	(−)	Monocytes	17.5	%
Total protein	6.7	g/dL	NSE	11.2	ng/mL	Anti‐titin Antibody	(−)			
Albumin	4.1	g/dL	HBs‐antigen	Negative		Anti‐zic4 Antibody	(−)			
LDH	146	U/L	HBs‐antibody	Positive		Anti‐GAD65 Antibody	(−)			
AST	21	U/L	HBc‐antibody	Positive		Anti‐Tr Antibody	(−)			
ALT	16	U/L	HBVDNA	Negative						
T‐bil	0.8	mg/dL	HTLV1‐antibody	Negative						
ALP	84	U/L	Anti‐nuclear antibody	< 40	Titre					
Sodium	138	mEq/L	s‐IL2R	325	U/mL					
Potassium	4.2	mEq/L								
Chloride	105	mEq/L								
Calcium	9.0	mEq/L								

*Note:* Serum PNS‐related antibodies were measured using immunoblot/line assay (EUROLINE Paraneoplastic Neurologic Syndromes 12 Ag (IgG), Euroimmun Japan, Japan).

Abbreviations: ALP, alkaline phosphatase; ALT, alanine transaminase; AST, aspartate aminotransferase; BUN, blood urea nitrogen; CEA, carcinoembryonic antigen; CSF, cerebrospinal fluid; CYFRA, cytokeratin 19‐fragments; KL‐6, krebs von den Lungen 6; LDH, lactate Dehydrogenase; NSE, Neuron‐specific enolase; proGRP, pro‐gastrin‐releasing peptide; s‐IL2R, soluble interleukin‐2 receptor; T‐bil, total bilirubin.

## 3 Discussion

This is the first report of anti‐Yo PNS in EGFR mutated NSCLC to our knowledge. The diagnosis of PNS requires the reasonable exclusion of alternative causes [3]. In our cases, neurological symptoms were confined to ataxia, which the distribution of metastatic brain tumours could not explain. Although leptomeningeal metastasis was present, nausea, headache, ventricular enlargement, or increased intracranial pressure were not observed. Anti‐Yo PNS has been reported to present with rapidly progressive pancerebellar ataxia and various brainstem‐related symptoms [2, 3]. Compared with other causes, anti‐Yo PNS closely matched the clinical presentation and met the criteria for probable PNS [3].

Almost all patients with Anti‐Yo PNS are female with ovarian or breast tumours [1]. They may rarely occur even in males, and are mostly associated with tumours mainly of gastrointestinal origin [2]. Reports of PNS associated with NSCLC are limited, and those involving EGFR mutated NSCLC are even rarer. Pozas et al. reported a case of anti‐Zic4 PNS in a patient with EGFR‐mutated NSCLC [4].

There is no established treatment for anti‐Yo PNS. Although immunotherapies, including corticosteroids, plasma exchange and IVIG, have been attempted, the reported efficacy remains inconsistent [2]. Antitumour therapy is recommended for PNS in general; however, when limited to anti‐Yo PNS, favourable results have not necessarily been obtained [2]. In this case, osimertinib was discontinued early due to ILD, so the efficacy of the antitumour therapy could not be assessed. Improvement in cerebellar symptoms was observed following steroid administration, suggesting the potential efficacy of immunotherapies.

A limitation of our case is the coexistence of multiple metastatic brain tumours and leptomeningeal metastasis, making it difficult to identify the precise cause of neurological symptoms. Impaired consciousness developed in the terminal stage may have been attributable to leptomeningeal metastasis. However, based on the clinical presentation and treatment course, the initial symptoms at diagnosis were strongly suspected to be related to anti‐Yo PNS. In the present case, the expression of Yo antigen in the tumour may have elicited an immunological response. However, Yo antigen expression in the tumour tissue could not be assessed. Moreover, an association between anti‐Yo antibodies and the EGFR molecular pathway has not yet been reported, hence further investigation is needed.

Importantly, NSCLC presenting with anti‐Yo PNS may harbour EGFR mutations. Treatment efficacy with EGFR tyrosine kinase inhibitors is anticipated, and further investigation is needed.

## Author Contributions

The first draft of the manuscript was written by Nobuhiro Okagaki, and all the authors commented on previous versions of the manuscript. All the authors have read and approved the final manuscript.

## Consent

The authors declare that written informed consent was obtained for the publication of this manuscript and accompanying images using the consent form provided by the Journal.

## Conflicts of Interest

The authors declare no conflicts of interest.

## Data Availability

The data that support the findings of this study are available on request from the corresponding author. The data are not publicly available due to privacy or ethical restrictions.
